# Quality improvement and antimicrobial stewardship in general practice – the role of the municipality chief medical officer. A qualitative study

**DOI:** 10.1080/02813432.2020.1794400

**Published:** 2020-07-31

**Authors:** Sigurd Høye, Anja Maria Brænd, Ivan Spehar

**Affiliations:** a Antibiotic Centre for Primary Care, Department of General Practice, Institute of Health and Society, University of Oslo, Oslo, Norway; b Department of General Practice, Institute of Health and Society, University of Oslo, Oslo, Norway; c Department of Health Management and Health Economics, Institute of Health and Society, University of Oslo, Oslo, Norway

**Keywords:** Quality development, antimicrobial stewardship, communicable disease control, drug prescription, professional autonomy, public health administration

## Abstract

**Aims:**

This study aimed to explore the conditions for the Municipal Chief Medical Officers’ (MCMOs) involvement in quality improvement in general practice, specifically concerning antibiotic prescribing practices.

**Methods:**

This qualitative study consisted of semi-structured in-depth telephone interviews and group interviews with MCMOs (*n* = 12). The interview guide aimed to explore the MCMOs’ views on their role and responsibilities regarding the quality of care in general practice. The data were analysed using systematic text condensation.

**Results:**

Three main themes were identified: 1) the relationship between the municipality and the general practitioner (GP), with the MCMO acting as an intermediary, 2) influencing the GPs’ work and 3) antibiotic use and infection control. The MCMOs perceived themselves as liaisons between the municipalities and the GPs. They emphasized building trust, showing respect and sharing common values in their interactions with the GPs, upholding the GPs’ professional autonomy. Working for quality improvement was considered a priority; however, MCMOs expressed a need for external support to establish a permanent quality improvement framework. The informants were positive about engaging in improving antibiotic prescribing practices because this combined the municipality’s responsibilities for quality improvement and communicable disease control.

**Conclusions:**

The MCMOs considered themselves as well-suited agents for quality improvement in general practice, as liaisons between the municipalities and the GPs. Quality improvement in general practice would benefit from a clearer structure in terms of the MCMOs’ roles and responsibilities. Within communicable diseases control, the MCMOs have a clear mandate, which places antimicrobial stewardship initiatives in a favourable position amongst other areas of quality improvement.

## Background

There is an ongoing focus on improving quality and patient safety in primary care [1,2]. General practitioners (GPs) constitute the main medical part of the primary care sector in Norway. They are contracted with the municipality and have certain obligations to provide available services as regulated through the Regular General Practitioner Scheme. In Norway, GPs are mainly self-employed and reimbursed through a combination of fixed payments per listed patient and a pay-for-performance scheme. GPs are themselves responsible for practicing within official guidelines and have no clinical superiors. However, the Municipal Health and Care Act states that each municipality must ensure that health care personnel have the necessary competence, and that health care service providers are expected to work systematically to improve patient safety and the quality of health care services [3]. The regulations for the Regular General Practitioner Scheme state that the municipality is responsible for ensuring such quality improvement work among GPs [4]. However, there is uncertainty among researchers and policy-makers regarding how this responsibility should be fulfilled, and the municipalities’ balancing act between surveillance and support is perceived as challenging [5]. The Norwegian Research Centre for Health Services has identified a need for more research on quality improvement in primary care [6]. Specifically, little is known about the conditions for the municipalities´ involvement in quality improvement in general practice.

All 356 Norwegian municipalities have at least one Municipal Chief Medical Officer (MCMO), who acts as the medical advisor to the municipality, and are assigned certain tasks as described in law or instructions [3]. Among these, the Communicable Diseases Control Act holds a special position, as it strictly defines tasks and responsibilities for the MCMO, i.e. to ‘contribute to effective measures to prevent infectious diseases and prevent them from being transmitted’ [7]. However, it has been argued that MCMOs are assigned unclear and comprehensive tasks that are not in line with their allocated resources [8].

There is an overuse of antibiotics in primary care [9], and avoidance of unnecessary antibiotic prescribing is regarded an important measure of quality of primary care. Quality improvement interventions towards GPs contribute to a more correct and lower use of antibiotics [10], but the broad implementation and maintenance of such antimicrobial stewardship interventions are challenging.

This study aimed to explore MCMOs’ views on their involvement in quality improvement in general practice in general, using antimicrobial stewardship interventions as a specific case.

## Methods

### Design and recruitment

This is a qualitative study consisting of interviews with municipal chief medical officers (MCMOs). MCMOs with responsibility for communicable diseases control were eligible for recruitment. We recruited two samples of informants: 1) A purposeful sample of MCMOs with a potential role as opinion leaders. We aimed for variation in gender and municipality size. Selected board members from ‘The communicable diseases control doctors’ (*Smittevernlegene*, an independent organization of MCMOs with responsibility for communicable diseases control) and the Norwegian Community Medicine Association (*Norsk samfunnsmedisinsk forening*, the community medicine speciality branch of the Norwegian Medical Association) were invited by e-mail to participate in a telephone interview. 2) A purposeful sample of MCMOs without any known role as opinion leaders. We aimed for variation in gender, geography and municipality size. The selected MCMOs were invited by e-mail to participate in a telephone interview. In addition, attending MCMOs at two communicable diseases control conferences, held bi-annually in each of Norway’s 18 counties, were invited by e-mail to participate in a group interview during or directly after the conference.

The recruitment and interviews were carried out simultaneously, and we ended recruitment when no new topics emerged in the interviews.

A total of 12 MCMOs were recruited: i.e. three in sample 1 and nine in sample 2. Sample 2 consisted of four individual interviews and two group interviews with two and three MCMOs, respectively. Table 1 presents the characteristics of the participants.

**Table 1.** Characteristics of the participants.

Characteristics	*N*
Total number of participants	12
Municipality size*	
Small (<5,000 inhabitants)	5
Medium (5,000–25,000 inhabitants)	5
Large (>25,000 inhabitants)	5
Gender	
Female	5
Male	7
Profession	
MCMO	8
MCMO and GP	4

* Total number exceeds 12 because some of the participants were MCMO for more than one municipality.

GP: general practitioner; MCMO: Municipal Chief Medical Officer.

### Data collection and analysis

The first author developed a thematic interview guide, which aimed to explore the MCMOs’ general views on their role and responsibility regarding the quality of care in general practice, and specifically their role and responsibility regarding the quality of antibiotic prescribing practices. The same interview guide was used for both individual and group interviews.

Seven individual semi-structured interviews and two group interviews were conducted by one of the authors (SH) between April and November 2016. The individual interviews lasted for an average of 26 min, while the group interviews lasted for an average of 36 min. All interviews were audio-recorded and transcribed in verbatim in Norwegian. The interviewing author reviewed the transcripts for accuracy. The recordings were handled confidentially, and the transcripts were anonymized. All interviewees received written information about the study and gave their oral consent to participation.

The data were analysed by two coders (SH and AMB) using systematic text condensation, which is a qualitative method for descriptive thematic analysis [11]. Firstly, we read all the transcripts to get an overview of the data and identify the preliminary themes, based on an inductive, rather than a deductive approach. We developed these preliminary themes separately, and they were subsequently discussed. Secondly, we identified and categorized the meaning units of the text and developed codes for these units based on the preliminary themes. The third step implied a systematic abstraction and summarization of the contents of each code group. The third author independently assessed the transcripts, coding units and summaries for consistency. Finally, we generalized the descriptions and concepts for each theme.

NVivo software was used to manage the data. The individual and group interviews were analysed together. Illustrative quotes were translated into English by the authors.

### Ethics

The participants gave their informed consent to the study. Data protection was approved by the Norwegian Centre for Research Data (NSD: 48136/3).

## Results

Three main themes with subordinate subthemes emerged through the analysis:

### The MCMOs were intermediaries in the municipality administration’s relationship with the GPs

All MCMOs experienced a good relationship with the GPs in their municipalities, as characterized by their mutual respect for each other’s roles. The MCMOs described their role towards the GPs as being organizers, supporters, advisers, dialogue partners and connecting links to the municipality administration. This followed from the management of GPs in the municipality where the MCMO was not the GPs’ superior or employer, but rather a contracting party, which also included professional development. One participant gave this description of the relationship:

If you get the message through that the municipality and the GPs actually are in the same boat regarding the fulfilment of requirements in the regulations for health-care services, and that the municipality in this context is a contracting party and not an employer, […] then a new relationship of trust has formed. (Informant 3)

Co-operation between GP representatives, the MCMOs, political decision-makers and the municipality administration was emphasized as important in achieving a teamwork culture in the municipality’s health care services:

It is really important that the GPs become part of the united health services in the municipality. I believe that is when the GPs will thrive and do their best, and we will get the best content in the services. The more the GPs are on their own running a shop, the less beneficial for the health-care services and the municipality. (Informant 2)

Only in a few instances, such as negotiating office rent, did the MCMO informants consider themselves as the GPs’ counterpart rather than their partner.

However, most of the informants knew about poor relationships between GPs and MCMOs. These relationships were typically in other municipalities or previously experienced in their own municipality, and their breakdowns were attributed to misconduct by the MCMOs or the municipality administration staff. Division between ‘them’ and ‘us’ (Informant 6), an emphasis on the municipality’s need for control (Informant 3), bureaucracy (Informant 1) and that the MCMO was not also a GP were given as explanations of the poor relationships.

Some differences emerged between small and large municipalities regarding the conditions for a good relationship between the MCMO and the GPs. In small municipalities, the MCMO typically had a combined position including clinical work as a GP. This dual role was emphasized as being important for the relationship because the MCMO knew the GPs’ world, spoke the same language, and were considered as one of their own (Group 1). One informant from a small municipality considered it necessary to have a GP background to be a MCMO.

It is common that the MCMO is not also a GP in large municipalities. Therefore, the MCMO needed to be conscious of their own conduct to achieve good collaborations with GPs; this was not to be taken for granted. Personal contact, respect (Informant 1) and support of the GPs rather than just inspections (Informant 3) were highlighted. Several of the informants had worked actively and intentionally to achieve good relationships over the years.

### The MCMOs were in a position to influence the GPs

The MCMOs considered themselves to be in a position to influence the GPs by means of professional supervision and facilitating quality improvement.

#### Professional supervision

The informants made a point to distinguish between arranging for quality improvement and monitoring the quality of the GPs’ work. They did not favour monitoring the GPs’ prescribing practices:

I have thought that it is important to not interfere and inspect whether GPs’ practice is sound and in accordance with laws and guidelines and the like. I have seen this as solely the responsibility of the County Governor. But it is the municipality’s responsibility to ensure that the GPs have internal control systems and so on. (Informant 1)

The informants considered this as a reasonable distinction for several reasons. Firstly, they did not regard the municipality as professionally superior to the GPs. Secondly, the public were mostly satisfied with their GPs, and the municipalities received few complaints, as one MCMO stated:

It would have been different if we had had a lot of complaints or dissatisfaction with the regular GP scheme. (Informant 7)

Thirdly, general practice was considered an especially independent service. Some informants reported that the GPs are highly motivated for practicing good medicine, and that good quality general practice health care services were therefore almost self-fulfilling. Others pointed out that the GPs had an independent responsibility to follow guidelines and deliver services according to best practice. The GPs were described as autonomous and not part of a hierarchy as in a hospital or in the municipality administration:

There is scarcely any hierarchy in a GP surgery; you don’t have a senior consultant as in a hospital. (Group2)

The informants were of the opinion that the municipality must respect the GPs’ autonomy:

I believe it is important that we understand the GPs’ autonomy. […] When we become part of the bureaucracy, you get a patronising attitude. […] We must consider the GPs as a resource, as the most competent group of health-care personnel we have in the municipality. (Informant 2)

Fourthly, many informants considered that professional supervision would undermine the relationship of trust between the MCMO and the GPs, which is a condition for working with quality improvement:

Many have taken a position of controlling and keeping an eye on the GPs. In my view, that means you lose the opportunity to support their quality improvement. (Informant 3)

#### Facilitating quality improvement

Working for quality improvement and patient safety was considered important. Comments were made that quality improvement and patient safety were highly prioritized in hospitals, and the same should apply to general practice.

The informants held that the municipality and the MCMO should play an active role in quality improvement in general practice. However, the informants generally experienced that the municipality administration did not take any initiative to perform these activities – the initiative had to come from the MCMO. The MCMO would generally be granted permission to engage in quality improvement initiatives, as long as this did not affect the MCMOs’ regular tasks. The MCMOs could prioritize themselves, with an underlying expectation of getting their priorities straight:

With me, I think they will say yes, that’s fine, it’s fine if you can give this priority. (Group 1)

The MCMOs appreciated this freedom, but many expected greater engagement from the municipality administration with regard to quality improvement. One informant asked for systems and funding to do quality work in general practice:

I believe the municipality should have systems for quality improvement with the GPs […]It needs to be approved so that there is acceptance for and maybe also some funding to do quality work among the GPs. I think that would give the municipality a lot in return. (Informant 2)

Hence, due to their lack of capacity, the MCMOa often did not give priority to quality improvement work, but rather reacted when problems emerged.

The interest towards quality improvement from the municipality administration depended on the size of the municipality. Both MCMOs in large municipalities and those who worked in both large and small municipalities pointed out that larger municipality administrations were more interested in quality improvement:

There is definitely an interest [in quality work] in the administration in [the large] municipality, in the other municipality the initiative is up to me (Informant 3)

Also, it was held that MCMOs who were employed full time would have better opportunities to engage in quality improvement than in small municipalities where the MCMO also worked as a GP.

### Quality improvement supported by an external framework

The informants were acquainted with different quality improvement tools for primary health care, and audit and feedback tools were highlighted. Some of the MCMOs had used such tools with their GPs; however, they expressed a need for external support to establish a more permanent framework for working with quality improvement and patient safety. A tool or system for quality improvement among the GPs was therefore encouraged:

If this could help us MCMOs to work with patient safety with the GPs, that might be the support we needed to get this into a system. (Informant 2)

The MCMOs who were particularly concerned with quality improvement tools were generally acquainted with these tools previously or they were particularly concerned about the municipality’s responsibility. They explicitly requested an easy-to-use toolkit to be offered them from some central entity.

[…] If each MCMO has to pull the load, it won’t happen, because there are no resources for this, there has to be a really simple tool. (Informant 3)

### Antibiotic stewardship – part of infection control responsibilities in the municipality

Antibiotic resistance was considered as a significant public health challenge. Several informants reported their experiences with antibiotic-resistant bacteria in the municipality as part of their work with infection control.

The MCMOs were concerned with the GPs’ antibiotic prescribing practices as they perceived this as part of their infection control responsibilities. Infection control was seen as a significant part of the MCMOs’ job, i.e. primarily managing disease outbreaks, but antibiotic use in the municipality was without objection included in the notion of infection control:

I think as a medical officer with responsibility for infection control, it would be neglectful not to engage with the antibiotic use in your own municipality. (Informant 6)

In this area, the MCMOs wanted more commitment from the municipality administration, both due to the municipality’s infection control responsibilities and to signals from central authorities.

Infectious disease control is high on the hierarchy in terms of legislation, but I have never experienced an interest from the municipality administration towards it. (Informant 4)

The MCMOs appreciated actions to improve antibiotic use, but they did not consider it to be their role to monitor GPs’ prescription patterns. In addition, the informants that were not GPs themselves were explicit about their own lack of clinical knowledge and experience in antibiotic prescribing practices.

## Discussion

### Summary of the main findings

Our findings indicate that MCMOs perceive themselves as being liaisons between the municipalities and the GPs. MCMOs emphasize building trust, showing respect and sharing common values in their interactions with the GPs. Furthermore, MCMOs are constrained by a lack of clear roles and responsibilities between the municipalities and MCMOs, and seem to be in favour of ensuring GPs’ professional autonomy.

Given these strategies and constraints, the MCMOs perceive themselves as being well-suited agents for quality improvement in general practice. This applies especially to antimicrobial stewardship efforts because they correspond with the municipalities’ responsibilities within communicable disease control. To implement quality improvement activities, the MCMOs request ready-made tool kits.

### Challenges facing municipalities’ involvement in quality improvement in general practice

Our study findings illustrate the contextual constraints facing both municipality administrations and MCMOs in their involvement in quality improvement in general practice. Firstly, the strong tradition of professional autonomy among GPs might limit municipalities’ and MCMOs’ ability to lead and oversee quality improvement initiatives in general practice. The challenge between management and professional autonomy in health care has been highlighted in the previous literature [12–14]. This has also been noted by GPs themselves in a recent study by Spehar et al. [15]. When discussing leadership challenges in general practice, the GPs described their struggles to find the balance between directing other colleagues and granting autonomy to them.

In our study, the MCMOs appeared to acknowledge the need for ensuring GPs’ autonomy, and focused on influencing GPs through building trust and showing respect for their professional values. This approach seems to be supported by a study by Spooner et al. [16], who looked at factors motivating British GPs to take part in a quality improvement scheme related to chronic disease management. Maintenance of professional autonomy and professional pride were reported as motivational factors. The authors concluded that substantial changes in clinical practice could result ‘when managerial vision is aligned to professional values’. In contrast, initiatives that are seen as unaligned with professional standards or values might be met with resistance. In concordance with the conclusion by Spooner et al. [16], our study might therefore suggest a need for stronger involvement of GPs in quality improvement initiatives, e.g. through existing formal meeting places between municipality administrations, MCMOs and GPs.

### How to ‘do’ quality improvement in general practice?

There has recently been an increased focus on leadership and organization in primary care in Norway [2], which has led to discussions on how to lead and organize the GPs. In a recent study that explored the role of Norwegian municipalities in managing the GP scheme [1], Bjørnhaug and Skyvulstad concluded that GPs should be led by someone with ‘a medical background, good knowledge of GPs’ work, and an ability to build good relationships with the GPs’. Nieuwboer et al. [17] echo some of these statements in a systematic literature review on the role of leadership in facilitating integrated primary care, which concluded that physicians appear to be the most adequate leaders, because of their hierarchical position in relation to other physicians.

A report from the Norwegian Research Centre for Health Services [6] emphasized the need for integrating national quality improvement directives into GPs’ daily practice, and the use of formal leadership structures to hold GPs accountable. The MCMOs in our study highlighted the need for more support from the municipality administrations; however, they did not express a wish to have a stronger or more formalized leadership mandate towards the GPs. Instead, they appeared to be in favour of preserving GPs’ professional autonomy. Accordingly, they made a sharp distinction between supporting the GPs in their quality improvement efforts and controlling the quality of their work, fearing that the latter would ruin the opportunity to bring about quality improvement. Based on our study, we suggest that quality improvement in general practice would benefit from a clearer structure in terms of the MCMOs’ roles and responsibilities, and that they appear to be ‘the right person at the right place’ for engaging in quality improvement in general practice as liaisons between the municipalities and GPs. This is in accordance with the views of the GPs, who request more engagement from the municipality [18]. The form of engagement might depend on the size of the municipality, as the role of the MCMO differs between small and large municipalities.

Our study highlights a seeming inconsistency; MCMOs look upon quality improvement as their responsibility, but they experience little support from the municipality, little capacity to do so, and no wish for a more professional leadership mandate. The study did not allow for further exploration of the MCMO’s views on how a medical leader role might be formed better to accommodate both GPs and society. However, the seeming inconsistency illustrates the challenges related to a lack of formal leadership over GPs, as well as to unclear guidelines for municipalities´ responsibility for quality improvement. These challenges should be addressed in future discussions about GPs and leadership roles in primary care.

### Prerequisites for quality improvement

Political commitment and adequate funding were identified as essential prerequisites for improvement in a Cochrane review of antimicrobial stewardship interventions [19]. This observation was in accordance with the expressed views of the MCMOs in our study. In addition, our informants requested ready-made quality improvement tools. Generally, such tools are based on audit and feedback of routine clinical data, which require that these data are readily available in an appropriate format [20]. The lack of easy access to clinical data in Norwegian general practice has been noted previously [21] and is seen as a barrier for professional development and delivery of care [2].

In settings with available quality improvement tools, the main challenge is the implementation of such tools [22]. Most research has focused on the effect of quality improvement on single GPs, but less on implementation at the organizational level. Future research should focus on identifying and assessing the effectiveness of strategies targeted at the wider context and organizational levels and examining the costs and cost-effectiveness of such implementation strategies [20]. Our results illustrate that both relevant tool kits, easy access to routine clinical data, and national systems for implementation should be in place for quality improvement to work.

### Antimicrobial stewardship

It has been argued that MCMOs are expected to take responsibility even in areas where they have no formal authority [23]. However, within communicable disease control, MCMOs have clear obligations under the Communicable Diseases Control Act [7]. All informants acknowledged antimicrobial resistance as a major societal challenge, and they regarded antimicrobial stewardship as an integral part of their responsibilities within communicable disease control. Hence, there is reason to believe that quality improvement initiatives in other areas than antibiotic use might not be equally favourably received among MCMOs.

Preliminary results from this study have been used to develop and tailor the content and implementation of an antimicrobial stewardship intervention towards GPs [24].

### Methodological considerations

The first author is the manager of a research project aimed at involving MCMOs in an antimicrobial stewardship intervention (the ENORM project [24]) and expected to find positive views on MCMOs’ involvement in quality improvement initiatives. The last author is involved in research on leadership in health care and expected to find challenges related to the lack of a formalized leadership structure between municipality administrations, MCMOs and GPS. We actively bracketed our own preconceptions during the analysis, and found views both in accordance with, and opposed to, our preconceptions.

All individual interviews were performed *via* telephone. Telephone interviews have generally been considered an inferior alternative to in-person interviews [25], but may also have advantages, such as allowing respondents to answer more freely. We used telephone interviews to achieve geographical diversity among the informants; however, some information, e.g. non-verbal communication, may have been lost through this approach. The authors did not transcribe the interviews themselves, but the interviewing authors reviewed all transcripts, and if in doubt on the correctness of parts of the transcripts, the audio file was compared to the transcript.

We intended to perform two focus group interviews with around five participants each at the county seminars; however, we only achieved the participation of two and three MCMOs in the two groups. The conversations were characterized by pronounced interaction between the participants, which is a prerequisite for focus groups [26]. Nevertheless, a group of two informants cannot be defined as a focus group; thus, we do not refer to these groups as focus groups.

The informants mostly agreed on the main findings. There were some differences between informants from small and large municipalities, as the former emphasized common ground as GPs, while the latter emphasized taking active steps towards GPs when building trust. All informants expressed positive views towards MCMOs’ involvement in quality improvement in general practice. However, negative views on such involvement may exist among the MCMOs who rejected the invitation to participate in this study.

## Conclusions

The MCMOs seem to fit well as facilitators of quality improvement efforts in general practice. However, quality improvement would benefit from a clearer structure regarding the distribution of responsibilities between MCMOs and the municipality administration. Ready-made quality improvement tool kits and national frameworks to facilitate implementation were welcomed by the MCMOs. Antimicrobial stewardship initiatives were considered to be in a favourable position because the MCMOs have a clear mandate within communicable disease control.
